# First Complete Genome Sequence of *Haemophilus influenzae* Serotype a

**DOI:** 10.1128/genomeA.01506-16

**Published:** 2017-01-19

**Authors:** Mariam Iskander, Kristy Hayden, Gary Van Domselaar, Raymond Tsang

**Affiliations:** a University of Manitoba, Winnipeg, Manitoba, Canada; b National Microbiology Laboratory, Public Health Agency of Canada, Winnipeg, Manitoba, Canada

## Abstract

*Haemophilus influenzae* is an important human pathogen that primarily infects small children. In recent years, *H. influenzae* serotype a has emerged as a significant cause of invasive disease among indigenous populations. Here, we present the first complete whole-genome sequence of *H. influenzae* serotype a.

## GENOME ANNOUNCEMENT

*Haemophilus influenzae* is a Gram-negative coccobacillus that can cause disease such as otitis media, pneumonia, and meningitis (1). *H. influenzae* can be classified into six serotypes (a to f) based on the characteristics of the surface polysaccharides; nonencapsulated strains also exist and are referred to as nontypeable *H. influenzae* (2). Historically, *H. influenzae* serotype b (Hib) had been the major cause of invasive disease and outbreaks worldwide until the development of a Hib conjugate vaccine in the 1980s (3). The introduction of the vaccine drastically decreased the incidence rate of invasive Hib disease; however, the vaccine does not protect against non–serotype b strains. Since the introduction of the vaccine, serotype f has replaced Hib as the most dominant invasive *H. influenzae* strain, while serotype a (Hia) has emerged as a significant cause of invasive disease among the indigenous populations of North America (4–7). Little else is known about Hia, and no publicly available Hia genomes have been reported to date. Here, we report the first whole-genome sequence of a *H. influenzae* serotype a strain (NML-Hia-1, isolated from a blood culture specimen).

Genomic DNA was extracted using the Qiagen DNeasy blood and tissue kit (Qiagen, Valencia, CA, USA). Whole-genome sequencing was performed with the PacBio RSII platform (Pacific Biosciences, Menlo Park, CA, USA) using a single-molecule real-time cell. The run generated 111,380 reads, with an average length of 9,889 bp and 576× coverage. The genome assembly was done using the Hierarchical Genome Assembly Process (HGAP) workflow, and the sequences were polished using Quiver (8) to produce a single 1,829,217 bp contig representing the closed, finished Hia chromosomal DNA with an average G+C content of 38.02% (Hia is not known to contain plasmids). The genome sequence was annotated using the NCBI Prokaryotic Genome Annotation Pipeline (PGAP, https://www.ncbi.nlm.nih.gov/genome/annotation_prok). The annotation predicted 1,643 coding sequences, 19 rRNA operons, and 59 tRNA genes.

The Hia genome size is relatively small in comparison to other publicly available *H. influenzae* genomes. The *H. influenzae* serotype d genome (Rd KW20; GenBank accession no. NC_000907) is only 0.92 kb larger than Hia, while serotypes f (KR494) and b (10810) (GenBank accession nos. NC_022356 and NC_016809) are 26.96 kb and 152.32 kb larger than Hia, respectively. The serotype f genome has 1,646 coding sequences, only three more than Hia, while serotype d has 1,610 coding sequences and serotype b has 1,853 coding sequences.

### Accession number(s).

The whole-genome sequence of *H. influenzae* NML-Hia-1 has been deposited in GenBank under the accession number CP017811.
